# Book Reviews

**Published:** 1908-04

**Authors:** 


### BOOK REVIEW

COSMETIC SURGERY.—The correction of featural imperfections. By Charles C. Miller, M. D.

Including the description of a variety of operations for improving the appearance of the face. 136 pages. 73 illustrations. Prepaid, $1.50. Published by the Author, 70 State St., Chicago, Ill.

This little book, perhaps, was not intended to cover the subject fully. In some points we find considerable detail, in others a marked superficiality. One can get a clue to many operations for the correction of real, or imagined, deformities, but scarcely enough information to warrant attempting the operations without further study and much experience. There is no reference to troublesome hemorrhage as might occur in operations about the mouth or in the nose. The subject of the electrolytic removal of hairs is carelessly treated. Parafin work is a bit more cautiously described. Some of the operations that vanity might image could well afford a field for much trouble and possibly damage suits. The description and treatment of some disease conditions are much too laxly given.

The illustrations average not very good. The price of the book seems excessive. However, the author gives some good points, and one could get a start for experimental work from its pages. Perhaps he may later produce a more perfect and complete work.

